# A Novel Runtime Algorithm for the Real-Time Analysis and Detection of Unexpected Changes in a Real-Size SHM Network with Quasi-Distributed FBG Sensors

**DOI:** 10.3390/s21082871

**Published:** 2021-04-19

**Authors:** Felipe Isamu H. Sakiyama, Frank Lehmann, Harald Garrecht

**Affiliations:** 1 Institute of Science, Engineering and Technology (ICET), Federal University of the Jequitinhonha and Mucuri Valleys (UFVJM), Teófilo Otoni 39803-371, Brazil; 2 Materials Testing Institute (MPA), University of Stuttgart, 70569 Stuttgart, Germany; frank.lehmann@mpa.uni-stuttgart.de (F.L.); harald.garrecht@mpa.uni-stuttgart.de (H.G.)

**Keywords:** structural health monitoring, FBG sensors, damage detection

## Abstract

The ability to track the structural condition of existing structures is one of the main concerns of bridge owners and operators. In the context of bridge maintenance programs, visual inspection predominates nowadays as the primary source of information. Yet, visual inspections alone are insufficient to satisfy the current needs for safety assessment. From this perspective, extensive research on structural health monitoring has been developed in recent decades. However, the transfer rate from laboratory experiments to real-case applications is still unsatisfactory. This paper addresses the main limitations that slow the deployment and the acceptance of real-size structural health monitoring systems (SHM) and presents a novel real-time analysis algorithm based on random variable correlation for condition monitoring. The proposed algorithm was designed to respond automatically to detect unexpected events, such as local structural failure, within a multitude of random dynamic loads. The results are part of a project on SHM, where a high sensor-count monitoring system based on long-gauge fiber Bragg grating sensors (LGFBG) was installed on a prestressed concrete bridge in Neckarsulm, Germany. The authors also present the data management system developed to handle a large amount of data, and demonstrate the results from one of the implemented post-processing methods, the principal component analysis (PCA). The results showed that the deployed SHM system successfully translates the massive raw data into meaningful information. The proposed real-time analysis algorithm delivers a reliable notification system that allows bridge managers to track unexpected events as a basis for decision-making.

## 1. Introduction

Transportation infrastructure plays a vital role in our society, as it enables people to engage in activities that produce private, public, and social benefits [1]. With relevance to large structure assets, bridge structures are built to connect people, shorten travel time, cross obstacles, improve traffic flow at complex crossroads, and allow access to regions otherwise inaccessible. In this sense, the socioeconomic impact of an inoperant bridge, as well as the life-threatening consequences of damage and unattended bridge network, can be incalculable. Therefore, the ability to evaluate the structural safety and the serviceability condition of existing bridge structures within the road infrastructure is one of the main tasks of engineers and a great desire of bridge owners. Additionally, many countries enforce the maintenance of their infrastructure assets with strict laws and regulations [2], such as the German model building code [3], the German civil code [4], and EU regulations for construction products [5].

A bridge is considered safe if the probability of failure during its service does not exceed a nominal value. The same is true for the serviceability, in which the likelihood of some service limits (e.g., vibration, deflection, etc.) to be exceeded must be small [6]. This concept has been widely adopted for decades in design codes throughout the world, and it is strictly observed during the design and construction phases of new bridges and other wide-span structures. However, to deem an existing bridge structure as safe or unsafe to operate is not as easy a task as it may seem.

On the one hand, the safety and serviceability requirements undergo constant changes regarding actions and resistance models, as well as the understanding of structural behavior and failure modes. As a result, generations of bridges that were designed using expired codes may be unsafe to perform, even if they are undamaged [7,8]. On the other hand, deterioration processes and changes in environmental conditions severely affect bridge structures’ safety and serviceability. Some examples are the reduction of resistance due to deterioration of concrete decks, corrosion or mechanical damage, the increasing traffic volume and vehicle weight [9], and the exposure to natural hazards due to climate change. Additionally, concrete material deterioration plays an essential role in the long-term damage accumulation and strength reduction in concrete structures [10]. For example, corrosion causes stiffness reduction [11], while cracking may increase the stress amplitude in the cross-section [12]. A comprehensive review about degradation models and stiffness degradation can be found in [11,13].

To cope with the challenges of managing existing bridge structures, the concept of bridge management systems (BMS) emerged to support engineers and bridge managers to provide cost-effective decisions for the planning of maintenance, rehabilitation, and replacement (MRR) [14]. A BMS is defined as the rational and systematic approach to organizing and carrying out all activities related to managing individual bridges within an infrastructure network [15]. It became well established in 1993 when the US government issued legislation outlining the obligatory requirements for a BMS model, as summarized by Tran (2018) [14]:

- Database (inventory, inspection data, maintenance data).
- Condition rating model (field evaluation of bridge condition).
- Deterioration model (prediction of condition of bridge components).
- Cost model (identification of costs and benefit).
- Optimization model (search of optimal MMR strategies).
- Risk model (risk ranking and risk assessment).

The different BMS practices among countries and companies have periodic visual inspection as the fundamental source of information [16]. The observed changes in the structure are recorded in a database and used as a qualitative condition rating. Although the visual inspections—when carried out regularly by qualified personal—are cost-effective and provide thresholds for the decision-making process, they provide little information about the inspected bridge’s actual structural safety and serviceability state without subsequent analysis and structural assessment. Moreover, the visual inspection procedures focus on visible physical damages, disregarding the safety and serviceability constraints adopted during the design phase. Hence, costly in-depth investigations and maintenance actions are often unnecessarily prompted based only on the visual perception of safety. Simultaneously, real threatening events can occur between inspection appointments or go unnoticed for not being visible or accessible, such as the failure of prestressed tendons under certain conditions and risks from faulty execution. Only in rare cases are the bridge maintenance actions triggered by a failed safety and serviceability check [6]. Therefore, visual inspections are inevitable as the primary means of information, but insufficient to satisfy the current needs for modern bridge maintenance programs [2,17,18] and societal expectations.

In this sense, the inclusion of non-destructive testing (NDT) and structural health monitoring (SHM) techniques into the bridge management process has become increasingly sought [19,20]. The possibility of estimating the actual load level and occurrence and detecting structural deterioration and damage events using SHM systems could bring the BMS to the next level of sophistication. With the information provided by the sensor network, more realistic numerical models can be achieved through structural identification [21] and model updating technics [22], and then be used, e.g., to simulate the real structural behavior and estimate its expected time-life. Moreover, a suitable number and placement of sensors and robust data acquisition software enable the real-life detection of damage events and the online triggering of alarms. 

The potential of SHM to optimize the management of bridge structures have motivated its research in the structural engineering field since the 1970s [23], with more than 17,000 papers published from 2008 and 2017. Nonetheless, the transfer rate of research to industrial practice is disappointing [24].

Cawley [24] notes that the scientific community should acknowledge the need to perform SHM research on real practical cases, rather than on simple beam and plate specimens with idealized failure modes, controlled environmental constraints, and without consideration of false calls. The deployment of full SHM systems, including data handling and decision-making, must be carefully designed. It is crucial to ponder, among others, the environmental conditions in which the system operates, the characteristics of the damages that the system should detect and their probability of occurrence, how the data is collected and transmitted, how the resulting data will be analyzed and translated into reliable performance indicators, and what actions are to be taken from the results and who will be responsible for making then [25]. Such considerations are difficult, if not unfeasible, to be investigated in simple laboratory tests.

Moreover, the sensor network and its operating system must allow the detection of local damages. Global monitoring, such as the exclusive use of vibration or shock sensors, has an excellent sensibility to detect changes in the boundary condition and mass distribution. Still, it may not be sensitive enough to detect local damage on large structures, depending on the setup and the system’s behavior. Cawley [26] reported that a crack with a depth of 1% of the cross-section height at the root of a cantilever beam would reflect a reduction of its natural frequency by less than 0.1%. Even if 10% of the cross-section were removed, the natural frequency would be reduced by less than 1%. Hence, the detection of local damage on bridge structures requires a high sensor-count network with an appropriate area coverage and signal-to-noise ratio to prevent false calls. Therefore, the system must be carefully designed to ensure its cost-effective deployment and meaningful operation.

An important aspect during the design of real-size SHM systems is the optimum sensor placement (OSP), which involves defining the minimum number of sensing points and sensor layout [27]. For simple reduced-scale models, the number of degrees of freedom (DOF) usually allows the placement of as many sensors as necessary to extract structural parameters correctly. However, in real-sized applications, structures may have many thousands of DOFs. At the same time, sensors can be placed at a finite number of locations [28], leaving a gap between the experimental SHM results and the real structural response [29]. While OSP algorithms based on modal analysis are well established [30], few researchers discuss OSP based on static parameters such as stress, strain, cracking, and long-term deformation [31,32]. Furthermore, different structure types require different OSP approaches. For example, in a plane truss, the correct estimation of the nodal displacements seems to be a reasonable OSP approach, which can be accomplished by measuring the axial deformation on selected truss elements [27,31]. On the other hand, in prestressed concrete bridges, the rupture of prestressed tendons at random locations may alter the structure’s static response, thus requiring a new sensor-layout to identify structural damages correctly. In the latter, SHM systems that prioritize distributed or quasi-distributed sensing are more appropriated.

Another issue of real-size SHM—often disregarded in laboratory tests—is the translation of the measured data to reliable information, i.e., the interpretation and handling of a massive amount of data [33]. A reasonable strain data collection on an average two-lane bridge concerning prestressed steel failure may result in hundreds of GB of data per month, corresponding to millions of spreadsheet lines. With the increasing availability of remote communication systems, the decreasing cost of sensors, and longer battery life and energy harvesting in remote locations, more and more structures are being monitored in some way. Still, the capacity to transfer a large amount of data into meaningful information has only marginally increased [34]. Due to the growing number of sensors, the data stream can become unmanageable; many SHM managers report that they do not know what information to keep, ending up with piles of hard disks to store TBs of measured data no one ever looks at in detail [35]. While in small deployments the engineer may individually analyze the data from installed sensors, a high sensor-count system must automatically highlight the anomalous signals where the existence, location, and severity of damages, followed by prognostics, is performed [36,37]. Moreover, the initial structural state is generally unknown outside of laboratory environments. The influence of temperature and other external disturbances, which can significantly influence the measured values, is also an example of drawbacks. Consequently, it is often necessary to manually check and interpret the data, requiring, on the one hand, the permanent availability of personnel and, on the other hand, leading to late detection or false alarms, which can compromise the monitoring system reliability.

Up to now, the detection of structural changes in SHM systems falls into two main philosophies: model-based and data-driven methods. The first, also known as model updating or the inverse approach, usually combines measured structural responses with finite element (FE) model predictions and supports long-term decision-making such as the lifetime and repair and evaluation [38,39,40,41,42,43,44]. However, FE modeling and updating are time-consuming and comes with a high computational cost. Given the many uncertainties, a calibrated model may not be correct even if its predictions match the measured observations [45]. For the data-driven methods, nonparametric approaches, such as the moving principal component analysis (MPCA) and robust regression analysis, can be applied to the data measurement history for damage detection and have been demonstrated in many laboratory tests and real-case applications [46,47,48,49]. Still, their real-time capability to detect structural changes is only feasible to a limited extent. The reasons lie in the unknown actual structural reaction due to local changes, such as prestressed steel rupture and crack formation, and the discrepancies between the real and theoretical properties related to the structure’s materials and geometry. Other methods independent of baseline references delivered promising results for real-life damage detection in bridge structures. They rely on statistical learning methods—e.g., neural networks and clustering—to extract intrinsic features without requiring prior knowledge of the structure health, and can be used on multiple sensing platforms, such as imaging technique, modal parameters, and static parameters (inclination, displacement, strain) [50,51,52,53,54].

This work presents a novel algorithm for the real-time analysis and alarm triggering of a high sensor-count monitoring system deployed on a structure that is subjected to environmental and random dynamic loading. The SHM system is based on a long-gauge fiber Bragg grating (FBG) (LGFBG) sensor network and was installed on a real-life prestressed concrete national highway bridge in Neckarsulm, Germany. Statistical and quantitative parameters are continuously updated from the strain and temperature data stream using a real-time computing (RTC) algorithm that performs, amongst others, a correlation analysis between adjacent sensors and allows the automatic detection of unexpected local structure changes by the minute. The algorithm is built based on redundancy to enhance its reliability and prevent false calls. A three-step check is performed to handle outliers, noise, and other random and unpredictable events. One important novelty of the proposed algorithm is that it was implemented inside the data acquisition software and is executed during runtime. In other words, the damage detection algorithm runs parallel to the measurements, and the analysis is carried out before data storage and data transmission take place.

Additionally, a post-processing method based on the principal components analysis (PCA) is applied to demonstrate the correlation coefficient analysis’s reliability to detect behavior changes in a high sensor-count system. Although the PCA post-processing results are compared with the real-time analysis results, they are two independent matters and should not be confused. The novel real-time damage detection algorithm does not depend on any post-processing evaluation. The PCA results are used to mathematically support the findings around the proposed algorithm.

The novel algorithm for real-time analysis and the post-processing method, are part of a pilot monitoring system developed to access the structural health of prestressed concrete bridge structures by providing robust, yet meaningful information to support bridge managers and bridge inspectors in the decision-making process of bridge maintenance. The system has a comprehensive data management design to handle the large volume of data produced from four different post-processing approaches and the real-time analysis algorithm.

The paper is organized as follows: Section 2 describes the monitored bridge and the installed monitoring system. Section 3 presents the data management system. Section 4 describes the novel real-time analysis algorithm and the post-processing method based on the MCPA. Section 5 contains the results of the real-time analysis and the MPCA post-processing method. Section 6 closes the work with the conclusions.

## 2. SHM System in Neckarsulm

### 2.1. Characteristics of the Monitored Bridge

**Figure 1:**
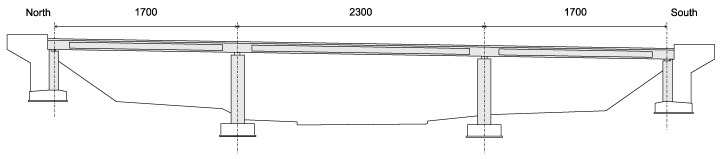
Longitudinal view of the bridge (dimensions in centimeters).

**Figure 2:**
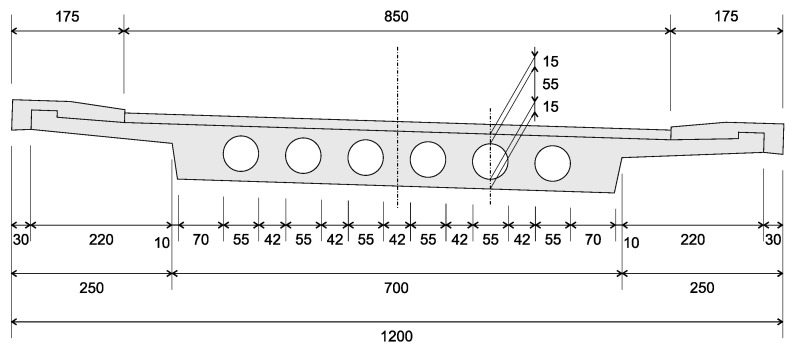
Bridge’s cross-section (dimensions in centimeters).

The monitored structure is a prestressed hollow-core concrete bridge constructed in 1964. The design load class is BK 60 (60 tons wheeled type load), according to DIN 1072. With a width of 11.08 m, it has three continuous spans with a total length of 57.00 m (17.00 m–23.00 m–17.00 m) without coupling joints (Figure 1 and Figure 2). The two center columns are designed as individual supports with pot bearing. The superstructure is supported by two linear rocker bearings on the southern abutment, and on the northern abutment, by two roller bearings. 

Like many of the prestressed concrete structures designed and built until the 1970s in Germany, the bridge was constructed with prestressing steel types St 145/160 Sigma (tensioning methods KA 141/40 and KA 35/10) which are known for their high vulnerability to stress-corrosion-induced cracking. A total of 18 tendons are placed in the total length, with an additional six tendons in the mid spam. In addition to the high increase in traffic loads compared to the year of construction in 1964, and the corrosion-induced cracking risk, other critical problems may arise due to construction methods and the design standards adopted back then [8]. From the structural point-of-view, the hollow-core bodies prevent two-axis load transfer and thus the redistribution of forces in the transversal direction. Likewise, shear forces and differential temperature loads were not considered to the extent that it is deemed necessary from today’s standards when the building was planned. Additionally, construction failures can already appear during construction caused by misplacement of the hollow-core bodies, and difficulties in compacting the surrounding concrete. Finally, the hollow-core cannot be examined as part of the regular visual building inspection, which means that any inside damage, especially the fill up with precipitation water, may not be detected in due time.

### 2.2. Characteristics of the Monitoring System

An elaborate fiber optic monitoring system based on long-gauge FBG (LGFBG) sensors were installed to continuously monitor strain and temperature changes, and vibration of the bridge superstructure. 

The strain monitoring consists of two parallel measuring lines, each with 27 LGFBG sensors connected in a series along the complete longitudinal direction, and five measuring lines in the shear direction, with five LGFBG sensors. Additionally, 10 LGFBG sensors are located on the sides of the main body to measure the strain at a higher position. For every LGFBG strain sensor, an embedded temperature sensor is present for temperature compensation on the fiber optic (FO). The LGFBG sensors’ brackets were mounted on the concrete’s surface using stainless steel hammerset anchors EA II M8 from Fischer, with a length of 30 mm to prevent damaging the rebars and stirrups (the bridge’s main deck has a concrete cover of 30 mm). 

Moreover, the concrete temperature is monitored at four different points at the bridge’s midsection, and the deck’s vertical acceleration is measured at two selected locations. The total of 184 sensors was divided into eight quasi-distributed arrays equipped with redundancy connection fibers, which enable measurements to be continued if a primary connection cable fails. The LGFBG sensors in this project were manufactured by Sylex s.r.o. [55], Bratislava, Slovakia, and the interrogator unit by HBM FiberSensing S.A. [56], Porto, Portugal.

The monitoring system in Neckarsulm has run continuously since November 2019 at a sampling rate of 200 Hz, generating over 70 thousand measurement points per second. The sampling rate was defined in order to optimize the representation of extreme values such as load peaks during the crossing of a vehicle. Considering that the average travelling speed at the bridge is 60 km/h (and there are speed cameras a few meters from the north abutment), a sampling rate of 200 Hz provides an 8-centimetre measuring step. This enables extracting the detailed dynamic behavior (e.g., peaks and strain influence lines), and characterizing the traffic load (vehicles’ average velocity, direction of travel, length, and number of axles), which were implemented in the SHM in Neckarsulm, but are outside the scope of this paper. Additionally, the high sampling frequency was chosen to depict sudden events, such as the rupture of prestressed tendons, which is the core of the novel real-time analysis algorithm. However, the high sampling rate does not imply that all the measured data must be stored at the same pace. For the novel damage detection algorithm, for example, the analyzed dataset rests in a temporary buffer. As soon as a batch of data is analyzed, only the statistical results are stored. The raw data is then discarded, except if the algorithm detects an anomaly, which, in this case, only the affected data segment is completely stored.

**Figure 3:**
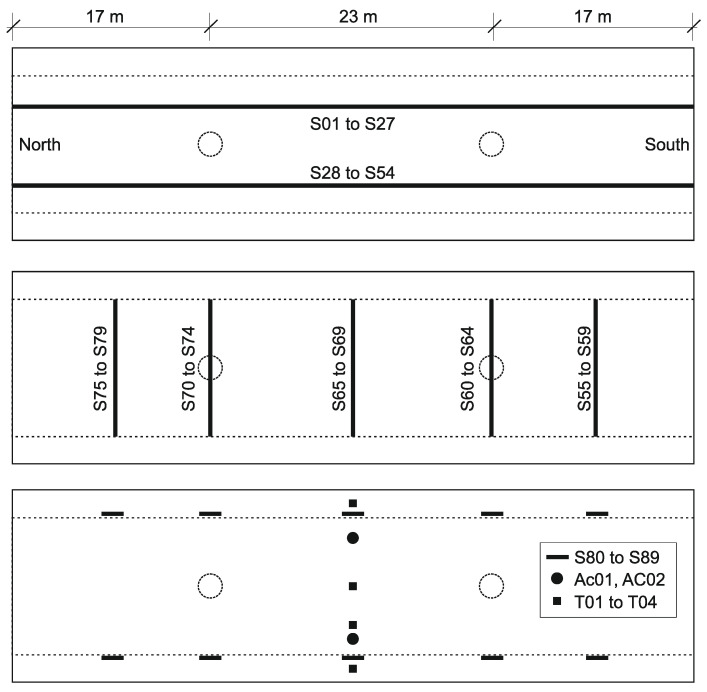
Schema representing the positioning of the sensors (bottom view of the superstructure). The two intermediate supports are indicated by dashed circles, the superstructure’s depth change by two dashed lines. Sensors S01 to S27 are the longitudinal LGFBG sensor line underneath the driving lane in the northern direction. Sensors S28–S54 are the sensors line underneath the driving lane in the southern direction. Sensors S55–S79 are the transversal sensor lines. AC01 and AC02 indicate the location of the acceleration sensors, T01–T04 indicated the temperature sensors.

**Figure 4:**
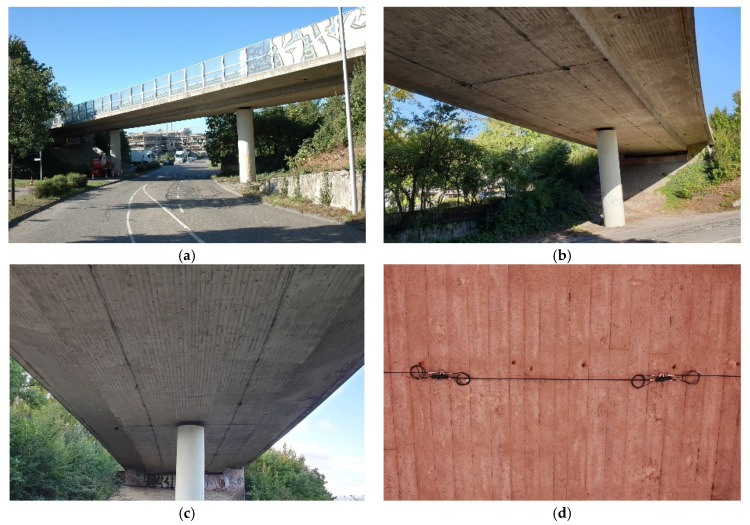
Overview of the bridge and the monitoring system: (**a**) Overview of the bridge; (**b**) view of installed sensors; (**c**) sensor distribution; (**d**) an LGFBG sensor with 2.05 m gauge.

A control cabinet was installed underneath the bridge, where the optical interrogator, the industrial computer for data acquisition, and the industrial LTE modem for data transfer are stored. A schema of the sensors is given in Figure 3, and overview photos are shown in Figure 4. The following sensors are installed on the structure:

- strain in the longitudinal direction (sensors S01–S54 and S80–S89): for monitoring in the longitudinal direction, two quasi-distributed strain sensor lines are attached to the bottom of the bridge, located in the area of the prestressed cables (the sensors S80–S89 were installed on the side of the structure at about 50 cm above the lower surface; the sensors S01–S54 have a gauge length of 2.05 m, and the sensors S80–S89 a gauge length of 0.50 m);
- strain in the transverse direction (sensors S55–S79): five strain sensor lines across the cross-section were installed on the underside of the bridge in the area of the maximum bending moments and on the two supports (these sensors have a gauge length of 1.35 m);
- temperature (sensors T01–T04): temperature sensors in the middle of the bridge, transverse to the direction of travel;
- acceleration (sensors AC01–AC02): two accelerometers with a vertical measuring direction in the middle of the main field, underneath each driving lane.

The FBG sensing technology is known for its high sensitivity, durability, and stability. It can provide quasi-distribute measurements over extensive measurement lengths, making it perfect for monitoring structures such as reinforced concrete and prestressed structures, where damage formation is usually local and random. The strain sensors have a precision of 1 µm/m and the temperature sensors of 0.1 °C. More information about the FBG sensors and fiber optic sensing in SHM of concrete structures can be found in [30,31,32,33,57,58,59,60].

## 3. Data Management

The monitoring system’s data acquisition is performed using the commercial software Catman AP developed and distributed by Hottinger Brüel and Kjaer (HBK), Darmstadt, Hesse, Germany. Beyond the required characteristics of a robust acquisition software, Catman also allows the online processing of computational channels and auxiliary channels that can be used to perform user-defined tasks via scripting.

**Figure 5:**
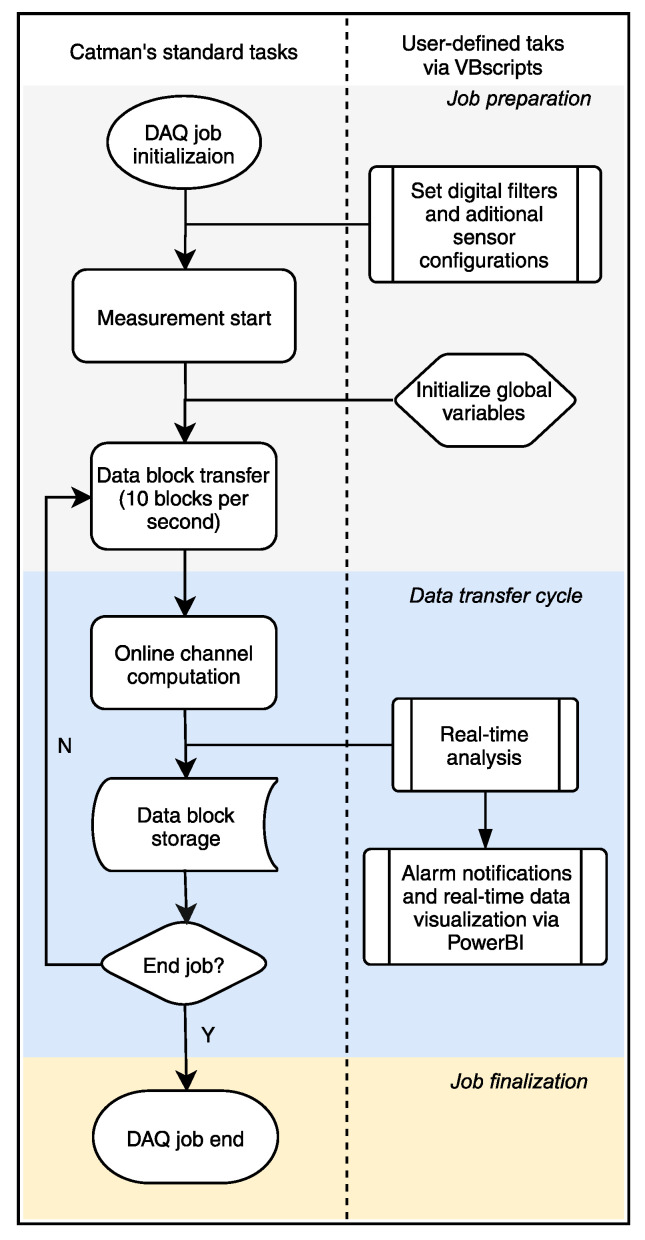
The basic workflow of a Catman’s DAQ job. The left side shows the system pre-defined steps. The right side shows the user-defined tasks performed via scripting.

The data acquisition (DAQ) process in Catman (DAQ job) can be divided into three main stages, namely the job preparation, the data transfer cycle, and the job finalization. During each step, a series of closed tasks are executed in the background without the user’s control. The basic workflow of a DAQ job is shown in Figure 5. However, using the Catman’s scripting functionality, it is possible to “intercept” a data block using a scripted procedure to carry out user-defined tasks. The system automatically sets the size of a data block according to the data sampling rate. For a sampling rate of 200 Hz, a data block has 20 measurement points for each of the 184 sensors, giving a total of 10 data transfer cycles per second per sensor (185 cycles every 100 ms). The data block cycle allows the implementation of RTC to process the data as it comes in, which is the core of the novel real-time analysis presented in this work. 

Moreover, the Catman software allows parallel data recorders configuration, where selected sensors with different saving configurations can be simultaneously stored into permanent files. For the monitoring system in Neckarsulm, four separate recorders were created and associated with specific events, namely the dynamic continuously event, the dynamic triggered event, the statistic journal, and the real-time analysis data. However, only the real-time analysis data belongs to the scope of this paper.

Given the high sensor-count and the dynamic measuring characteristics, the amount of data generated is enormous; thus, it was necessary to create a robust data management for both the data storing and the post-processing. The chosen solution was integrating the software MathWorks MATLAB [61], Natick, MA, USA, and the MySQL [62], Austin, TX, USA, database using scripts written specifically for this application. 

**Figure 6:**
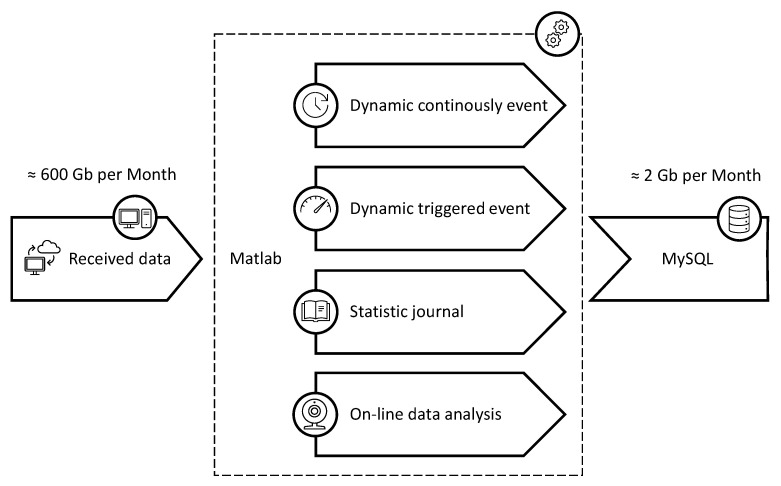
Data storing workflow for the parallel recorders.

MATLAB is a powerful analysis software known for its vast availability of mathematical and statistical pre-generated methods for data analysis and numerical computation. MATLAB allows the connection with a SQL database and the execution of queries inside the MATLAB workspace among its many attributes. The data on MATLAB can be stored in a SQL database, and an SQL database can be loaded into MATLAB for analysis. The SQL database can save millions of data entries and provides fast access to a specific set of data within the database, optimizing the data saving and the data query for analysis. Figure 6 shows the data storing process, where the received monitoring data is saved into a MySQL database through MATLAB processing. The monitoring system generates about 600 GB of raw data every month. Only 2 GB are effectively stored in the SQL database after executing the pre-processing scripts developed exclusively for this project. Nevertheless, all raw data generated during this SHM project is being stored in an external hard drive disk (HDD) for scientific purposes.

The SQL database was structured to allow easy management and query for post-processing and data visualization. The database schema has four groups of relational tables divided by the type of recorder, as shown in Figure 6, and a single table to store the sensors’ information and calibration coefficients. Additionally, a rainflow analysis result from the dynamic continuously event is saved separately as a MATLAB structure after its pre-processing. 

**Figure 7:**
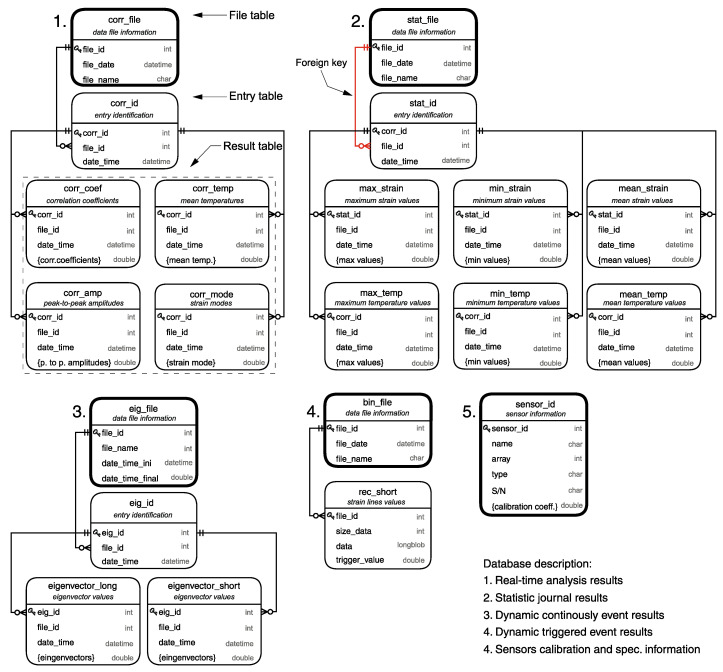
SQL schema and tables.

Figure 7 shows an overview of the SQL schema and its table groups. Each group has a parent file table that records the files’ information and gives them a unique file-id number, followed by a child entry table that indexes all the entries in each file with a unique entry-id number. Finally, the records are organized in separate result tables, where each row corresponds to a unique entry-id. The fields closed by curly brackets {} in the result tables refer to an array of fields, usually one for each sensor, and is represented this way for simplification.

The tables within each group are linked together by foreign keys, where the entry-id in the result tables refers to the entry table, which directs its file-id to the files table. The SQL relational structure optimizes the data-selection from the database without the loss of referential integrity and facilitates database management. The JOIN clause, e.g., permits the rows and columns from two or more result table to be combined based on their related entry-id, allowing the selection of result from the desired period of a specific sensor without the need to load the entire dataset. Moreover, if a file-id must be removed from the file table, all the rows in the entry table related to that file are automatically deleted. Likewise, all the rows in the results table linked to the removed rows in the entry table are erased. 

## 4. Theory and Methodology

### 4.1. Real-Time Analysis Algorithm

For the installed SHM system in Neckarsulm, an algorithm was developed and implemented to determine whether unexpected changes have occurred in the structure based on the real-time analysis of the measured strain and temperature data. The algorithm is registered for a patent. Statistical values are continuously updated from the strain and temperature data stream of each sensor over an optimized *n*-sampled moving time window $\tau_{n}$, namely the statistical strain mode *Mo*, the arithmetic strain mean *μ*, the arithmetic temperature mean $\bar{T}$, and the maximal peak-to-peak amplitude *u*. Additionally, the strain data from the time window $\tau_{n}$ for every two adjacent sensors p and q are analyzed, where the correlation coefficient $\rho_{pq}\left(\tau_{n}\right)$ for the measured strain $s_{p}\left(k\right)$ and $s_{q}\left(k\right)$ are calculated according to:

(1) $$\rho_{pq}\left(\tau_{n}\right)=\frac{\sum_{k=1}^{n}\left(s_{p,\;\;k}-\mu_{p}\right)\cdot \left(s_{q,k}-\mu_{q}\right)}{\sqrt{\sum_{k=1}^{n}{\left(s_{p,k}-\mu_{p}\right)}^{2}}\cdot \sqrt{\sum_{k=1}^{n}{\left(s_{q,k}-\mu_{q}\right)}^{2}}}$$

For a continuous monitoring system with high sampling rate measurements on coherent structures with consistent loading, the correlation coefficient between a pair of sensors must remain constant and close to one, if they are well correlated, or close to zero, if there is no correlation. In the case of adjacent sensors disposed along the longitudinal direction of a continuous beam, the correlation coefficient should remain stationary and close to one until a change occurs in the structural system [63]. The evaluation of the correlation between two sensors can also be used to infer already existing geometric discontinuities, e.g., hollow bodies or built-in parts, or pre-existing damages in the structure.

Although the correlation coefficient is a relevant parameter, it cannot be used alone as an indicator of structural change since noise and influences from wind or traffic loads, amongst others, can also lead to deviations in the correlation. Therefore, the implemented algorithm is based on a three-step validation to avoid false calls and enhance the system’s reliability. Should the correlation coefficient for two correlated sensors drop below a pre-defined threshold within the time window $\tau_{n}$, the maximal peak-to-peak amplitude *u* and the strain-offset through the statistical mode *Mo* of both sensors during $\tau_{n}$ are examined. Under normal operation conditions, the peak-to-peak amplitude is directly related to the traffic load. Simultaneously, the statistical mode represents the strain signal offset due to the environment temperature variation and can be considered the “unloaded state” of the bridge for a short time window.

**Figure 8:**
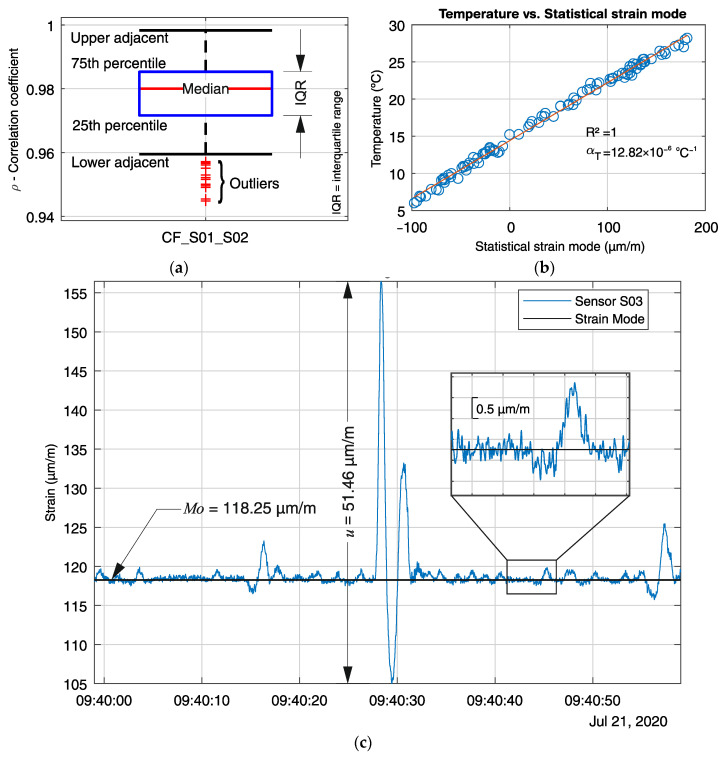
Statistical parameters: (**a**) Box plot of the correlation coefficient between sensors S01 and S02 measured from 16 July to 6 November 2020; (**b**) correlation between the strain mode and the temperature from sensor S01 measured from 14 July to 6 November 2020; (**c**) strain signal from S03 for a one-minute time window, showing the strain mode, and the peak-to-peak amplitude.

Suitable limit values must be defined for all three indicators. The correlation coefficient threshold is set as 0.9, which is a relatively low value considering the observed measurement history for one year. Figure 8a, e.g., shows the box plot for all measured correlation coefficients between sensors S01 and S02 from 14 July to 6 November 2020. It can be observed a median of 0.98 and narrow interquartile range, with all values above 0.94. The peak-to-peak amplitude can be determined by an initial load test or estimated from the strain history over a long period. Since no load test was performed on the monitored bridge, the peak-to-peak amplitude limit was taken from the cumulative distribution plot of the maximal observed values for the sensor S14, located at the middle of the bridge, and set as 60 µm/m. The strain offset is defined by observing the statistical mode of short moving time-windows $\tau_{n}$ over a long measuring period. Figure 8b shows the correlation between the statistical mode and the mean temperature calculated for each time-window $\tau_{n}$ from 14 July to 6 November 2020. Since the strain sensors are temperature compensated, and the bridge superstructure is free to deform in the longitudinal direction, the changes in the mode values are mainly due to the structural deformation due to the temperature variation. A linear correlation between the strain mode and the temperature can be observed, with a statistical variation coefficient of one and an inclination of 12.82 × 10^−6^ °C. Therefore, an abrupt non-linear behavior between the strain mode and the temperature variation indicates an unexpected event unrelated to temperature changes, which could for example be due to a crack opening or a change in the static behavior.

Figure 8c shows the strain signal from sensor S03 during a one-minute time window $\tau_{n}$. The recorded event occurred on 21 July 2020, starting at 09:39:59.000 h and ending at 09:40:58.995 h. The calculated strain mode of 118.25 µm/m is marked with a horizontal line, and the peak-to-peak amplitude of 51.46 µm/m is displayed as a dimension line. A segment of the strain signal was enlarged to show the quality of the signal and the precision of the mode to estimate the signal offset when short periods are analyzed. It can be seen that the signal noise amplitude is less than 0.5 µm/m, and the mode line is a fair representation of its relative midline.

**Figure 9:**
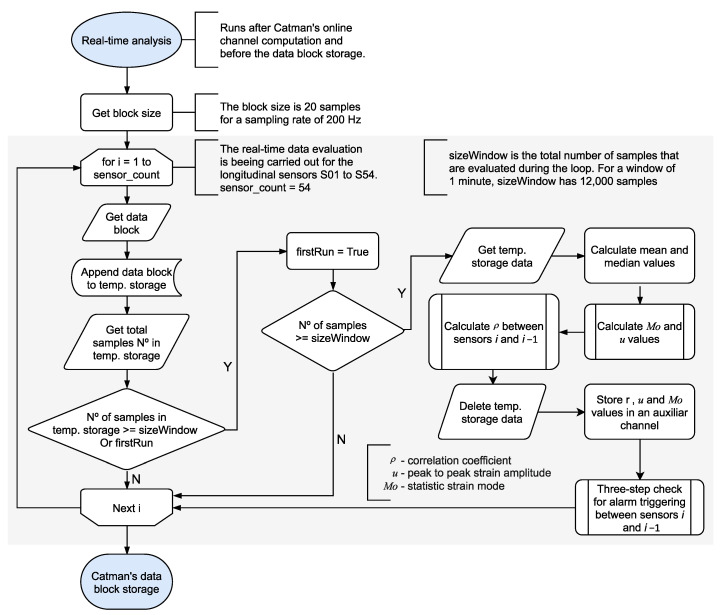
Real-time analysis subprocess flowchart (corresponds to the data transfer cycle in Figure 5).

**Figure 10:**
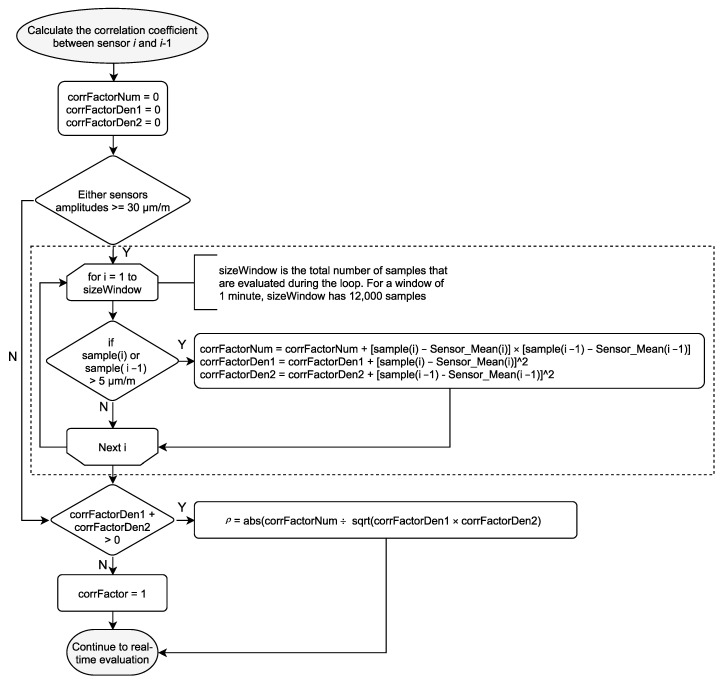
Correlation coefficient calculation subroutine flowchart (refer to Figure 9).

As shown in Figure 5, the real-time analysis intercepts the data block transfer containing each sensor’s last 20 measured samples. Figure 9 shows the flowchart of how the algorithm handles the data block for each sensor *i* during the real-time analysis. The data block’s samples are collected and appended in a temporary buffer until the size of the temporary buffer reaches the size of the moving time window $\tau_{n}$. When the temporary buffer’s size equals the $\tau_{n}$ size of 12,000 samples (equivalent to 60 s), the statistical parameters are extracted from the temporary buffer dataset, as explained at the beginning of this section. Next, the correlation coefficient between sensor *i* and sensor *i* − 1 is calculated, as Figure 10 shows. The correlation coefficient is calculated if the peak-to-peak amplitude of either sensor is higher than 30 µm/m, and a low-cut filter removes samples with an absolute value inferior to 5 µm/m. After the calculations, the temporary buffer is erased, and the statistical parameters are sent to auxiliary channels for permanent storage. It is important to note that the algorithm runs during runtime inside the data acquisition software. Therefore, all the tasks take place parallel with the measurements, and before data storage and data transfer.

The 30 µm/m limit was set to optimize the computational performance. In theory, each pair of sensors’ coefficient could be calculated every minute, regardless of the strain amplitudes. However, the authors chose not to overload the processor with small strain values that are known to have no impact on structural integrity. The 5 µm/m low-cut filter is meant to remove noise. Take, for example, the correlation coefficient between two sensors, where only one heavy vehicle crossed the bridge during a 60 s time window. Considering that it takes about 3.5 s for a vehicle with a speed of 60 km/h to cross the bridge, there would be 700 samples (6%) where the strain values between the sensor would be well correlated, and the remaining 11,300 would be just noise.

To set the time window’s size, one must find the equilibrium between computing performance, reaction time to detect damages, and influence of the strain signal caused by the traffic loading for the analyzed period. If the time window is too short, the algorithm must run often, and there is a risk that the system will overload. Likewise, the impact of abrupt strain variations in the correlation analysis reduces as the time window increases and the reaction time to detect damages increases.

**Figure 11:**
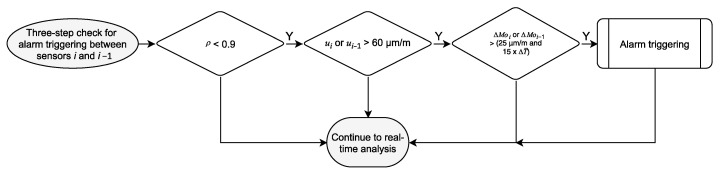
Three-step check subroutine for alarm triggering flowchart (refer to Figure 9).

Finally, the three-step alarm trigger checks the calculated parameters for unexpected behavior during the last 60 s dataset, as shown in Figure 11. If all three-steps are triggered, an alarm is immediately sent to the bridge managers. As soon as a time window is thoroughly analyzed, the process repeats itself from the beginning for the next set of 12,000 samples.

In contrast to the traditional alarm triggering approaches, the monitoring system in Neckarsulm does not rely on absolute or singular thresholds. Each derived parameter is particularly sensitive to different factors: the statistical strain mode to the temperature influence, the peak-to-peak strain amplitude to the traffic load, and the correlation coefficients to the static system behavior. Only if the three indicators individually show critical values, an alarm is triggered, allowing the bridge managers to evaluate all three indicators together with the complete measurement data from all sensors. The proposed system allows the detection of unexpected events by the minute and a post-processing of the acquired data for long-term analysis of the structural integrity and life expectancy.

### 4.2. PCA Method

The correlation coefficient analysis is a powerful method to visualize the relationship between two variables and measure their linear dependence. However, in a multivariate statistics problem, it is difficult to visualize the relationship between multiple variables and determine their contribution to the driving principle that governs the system’s behavior. The principal component analysis (PCA) is a quantitative method used to simplify such problems by replacing the original data with a new set of variables that still contains most of the information, called the principal components [61].

Although the PCA post-processing is implemented in the SHM system in Neckarsulm, it is not part of the proposed real-time damage detection algorithm. Namely, the proposed real-time algorithm is independent of the PCA analysis. However, this paper shows the PCA implementation and results to support the proposed real-time damage detection algorithm with a mathematical background, especially regarding the correlation coefficients’ reliability on detecting structural changes.

The principal components have no physical meaning, but they describe the directions that explain a maximal amount of variance, i.e., the axes that provide the best angle to see and evaluate the data. The first principal component is composed of the axes’ directions that capture each variable’s largest possible variance. The second principal component is another set of axes perpendicular to the first and accounts for the next highest variance. This process continues until the number of calculated principal components equals the number of variables in the original data. The full set of principal components is a square matrix of order *n*, where *n* is the number of variables. However, it is commonplace that the first few principal components explain over 80% of the total variance. Therefore, they can be used to understand the driving forces that generated the original data [64].

The principal components are constructed by calculating the eigenvectors and eigenvalues of the data’s covariance matrix. The eigenvectors represent the direction of the axes where there is the most variance, while the eigenvalues are coefficients that give the amount of variance carried by each eigenvector. The principal components are simply the eigenvectors sorted in order of their eigenvalues.

Therefore, to evaluate each variable’s contribution (sensor) in the overall structural system’s behavior, a PCA analysis is performed to calculate the principal components from the sensors’ strain history. Given the high number of sensors, the PCA is achieved by dividing them into groups of sensors, sorted by measurement line and span (Figure 3) to allow a proper assessment of the variables’ dependency, as follows:

- group A: sensors S01–S27; and
- group B: sensors S28–S54.

First, a matrix with the strain histories from all sensors in a group is constructed for the analyzed time window $\tau_{n}$, where the result is a *N* by *n* matrix:

(2) $$S\left(\tau_{n}\right)=\left(\begin{array}{ccc} S_{i}\left(t_{j}\right) & \cdots & S_{N}\left(t_{j}\right)\\ \vdots & \ddots & \vdots \\ S_{i}\left(t_{j+n-1}\right) & \cdots & S_{N}\left(t_{j+n-1}\right) \end{array}\right)\;,\;\mathrm{with}\;i=1\;\mathrm{to}\;N\;\mathrm{and}\;n=size\left(\tau_{n}\right)$$

where *N* is the number of sensors, *j* is the first sample in the time window $\tau_{n}$, and n is the time window’s size in samples. Since the PCA is sensitive to the initial variables’ variances, the resulting matrix must be normalized to assure that they will contribute equally in the analysis. A normalization is used, where each variable is centered on having a mean of 0 and rescaled to have standard deviation 1. The normalized matrix s of the strain history is given as:

(3) $$s=\left(\begin{array}{ccc} S_{i}\left(t_{j}\right)-\bar{S_{i}\left(\tau_{n}\right)} & \cdots & S_{N}\left(t_{j}\right)-\bar{S_{N}\left(\tau_{n}\right)}\\ \vdots & \ddots & \vdots \\ S_{i}\left(t_{j+n-1}\right)-\bar{S_{i}\left(\tau_{n}\right)} & \cdots & S_{N}\left(t_{j+n-1}\right)-\bar{S_{N}\left(\tau_{n}\right)} \end{array}\right)$$

where $\bar{S_{i}\left(\tau_{n}\right)}$ is the mean value for the sensors’ strain history during $\tau_{n}$ Next, the covariance matrix $C\left(\tau_{n}\right)$ for all measured samples is constructed from the standardized matrix s as follows:

(4) $$\begin{array}{c} C\left(\tau_{n}\right)=\left(\begin{array}{ccc} cov\left(s_{1},s_{1}\right) & \cdots & cov\left(s_{N},s_{1}\right)\\ \vdots & \ddots & \vdots \\ cov\left(s_{1},s_{N}\right) & \cdots & cov\left(s_{N},s_{N}\right) \end{array}\right)\\ \mathrm{with}\;cov\left(s_{p},s_{q}\right)=\frac{1}{n-1}\sum_{k=1}^{n}\left(s_{p,\;k}-\mu_{p}\right)*\left(s_{q,\;k}-\mu_{q}\right)\;,\;\mathrm{with}\;p\;\mathrm{and}\;q\;=\;1\;\mathrm{to}\;N \end{array}$$

where $\mu_{p}$ is the mean of the data series $s_{p}$, $\mu_{q}$ the mean of the data series $s_{q}$, and * denotates the complex conjugate. Finally, the eigenvalues $\lambda_{i}$ and eigenvectors $\Psi_{i}$ of the covariance matrix are obtained by satisfying the equation:

$$\left[C\left(\tau_{n}\right)-\lambda_{i}\;I\right]\;\Psi_{i}=0,\;\mathrm{for}\;i\;=\;1\;\mathrm{to}\;N.$$

When their eigenvalues sort the eigenvectors’ eigenvalues in decreasing order, they are arranged in order of significance, resulting in a *N* × *N* matrix, also called the principal components (PC) matrix, where *N* is the number of variables. As explained before, the first few columns contain most of the information about the original data variance. They can be used to understand how each variable contributes to the overall behavior and how the variables interact with each other.

## 5. Results from the Real-Size SHM

In this section, the detailed results from the novel real-time analysis for the sensors S02, S03, and S04 is first shown, followed by the general study of sensors S01–S27, located in one of the longitudinal quasi-distributed measuring lines. The real-time evaluation algorithm is based on a three-step validation process. Three statistical parameters are continuously calculated parallel to the reception of measurement data for all longitudinal direction sensors (S01 to S54). Each step will be referenced as a filter, as summarized in Table 1. As described in Section 4, the strain correlation coefficient *ρ* between neighboring sensors, the maximal peak-to-peak amplitude *u*, and the statistical strain mode *Mo* are determined for every one-minute time window $\tau_{n}$. Lastly, The PCA results are used to evaluate the correlation between the sensors and their contribution to the structural system’s behavior.

### 5.1. Real-Time Analysis Algorithm

**Figure 12:**
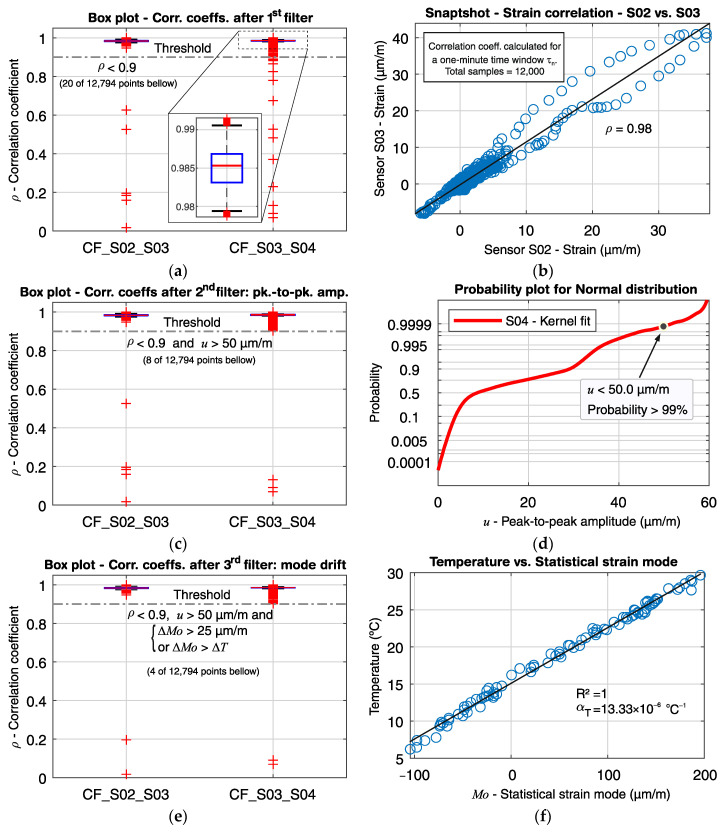
Three-step real-time analysis demonstration—sensors S02, S03, and S04: (**a**) Correlation coefficients and points below the threshold after the first filter. The zoom window shows the amplified box plot for the correlation coefficients between sensors S03 and S04; (**b**) example of the correlation coefficient between sensors S02 and S03 during a one-minute time window with 12,000 samples; (**c**) correlation coefficients below the threshold after the second filter; (**d**) probability plot for the peak-to-peak amplitude *u* for sensor S04; (**e**) correlation coefficients below the threshold after the third filter; (**f**) correlation between the statistical strain mode *Mo* and the temperature for sensor S03. Analyzed data period: from 14 July to 6 November 2020.

Figure 12a shows the box plot for the strain correlation coefficients *ρ* between sensors S02 and S03 (CF_S02_S03), and sensors S03 and S04 (CF_S03_S04), where a total of 12,794 moving time windows $\tau_{n}$ were recorded from 14 July to 6 November 2020 (116 days). It can be noticed that both pair of sensors have high correlation magnitudes, both with medians of approximately 0.98, and narrow interquartile and score ranges. The number of outliers is about 10% of the total cases, which is expected in large sample sets. The A magnified box plot for CF_S03_S04 is given in Figure 12c, where the median, interquartile range and extreme limits are depicted in detail. Figure 12b shows an example for the dependency between the strain signals from sensors S02 and S03 during a time window $\tau_{n}$, with the calculated correlation coefficient *ρ* = 0.98.

When the first filter is applied, a total of 20 outliers representing 0.156% of the total measured points remain below the first filter’s threshold (*ρ* < 0.9). In other words, the correlation coefficients smaller than 0.9 are treated as potentially problematic, as they indicate a loss of linearity behavior between two neighboring sensors that ought to be otherwise linearly correlated and should thus be further analyzed. If only the first filter were used for the real-time analysis, the bridge managers would have received 20 alarm calls—around one call per week—for just two pairs of sensors. They would not have had additional information to judge whether the alarms were related to unexpected structural integrity changes or false calls (e.g., noise).

Next, the second filter is applied, where the peak-to-peak strain amplitudes *u* of each sensor are checked at the time-intervals corresponding to the detected small correlation coefficients that went through the first filter. The peak-to-peak strain amplitude of a short moving time-window, such as $\tau_{n}$, is closely related to the traffic load; hence, values within the normal range of traffic operation can be disregarded during the real-time analysis, and only those with values above a specified limit should continue to be treated further. In this example, a peak-to-peak amplitude limit of *u* > 50 µm/m is applied for demonstration purposes (during operation, the limit is set at *u* > 60 µm/m, given that, based on the entire measurement history, there is a small than 1% probability that the peak-to-peak amplitude will not exceed 60 µm/m). Figure 12a shows the box plots for the correlation coefficients after removing the points that passed the first filter (*ρ* < 0.9) but were retained at the second filter for *u* > 50 µm/m. Only eight remained after the second filter from the 20 points below the first filter’s threshold. In Figure 12d, the cumulative distribution function of the peak-to-peak strain amplitude is depicted. It can be noted that the adopted limit for *u* is within the service traffic load for the measurement history, where there is a probability less than 1% that *u* will exceed 50 µm/m.

Even though the number of distinguished points was considerably reduced after the second filter, there is still insufficient information to call the remaining points problematic. A peak-to-peak strain amplitude above the average traffic operation does not necessarily mean that structural damages took place. The safety design checks require that the ultimate loads be higher than expected service loads. 

Finally, the third and last filter is applied. In this stage, the strain mode *Mo* at the time window $\tau_{n,i}$ for each remaining point *i* is compared with the strain modes of the time windows $\tau_{n,\left(i-1\right)}$ and $\tau_{n,\left(i+1\right)}$. Given that each strain sensor has its own temperature sensor for temperature compensation, the drift in the strain signal offset over time can be related to the structure’s deformation due to temperature variation. Thus, the signal offset of each sensor for the time window $\tau_{n}$ can be determined from the statistical strain mode. Since the traffic load is intermittent, and the crossing of a vehicle takes a few seconds, the statistical mode for a short time window should represent the signal offset for an “unloaded state”. The correlation between the strain mode (signal offset) and the temperature at sensor S02 is shown in Figure 12f. A linear correlation can be observed, with *R^2^* ≈ 1 and $\alpha_{T}=13.33\times 10^{-6}\;{}^{\circ}C^{-1}$. Therefore, the strain mode drift should not be higher than the expected deformation due to the temperature variation for that same period, when analyzing two consecutive short-time periods. Thus, if the strain mode variation $\Delta M_{0}$ is higher than 25 µm/m (which is equivalent to a crack opening with a width of 0.0125 mm) and $\Delta M_{0}>15\;\text{µ}m/m\cdot K\times \Delta T$ (*Mo* in µm/m and temperature in °C), the point is called as problematic, and the system sends an alarm to the bridge managers.

Figure 12e shows the remaining points after applying the third filter, where four points remain after the application of the three-level filtering. These four points resulted from a demonstration during a visit at the bridge of the real-time analysis operation. A small weight was hanged on the gauge-length of sensor S03, causing a rapid perturbation on its measurement signal. Since the neighbor sensors S02 and S04 were not affected, a loss of linearity between sensor S03 and its neighbors was detected by the correlation coefficients CF_S02_S03 (*ρ* = 0.196) and CF_S03_S04 (*ρ* = 0.10), thus triggering the first filter (*ρ* < 0.9). The slight change in the gauge-length curvature due to the added weight also produced an immediate peak-to-peak amplitude of 640 µm/m, which triggered the second filter (*u* > 60 µm/m). Finally, after the initial perturbation caused by the hanging of the weight, the system went back to equilibrium, and the strain mode from sensor S03 suffered a drift of 222 µm/m (equivalent to a crack opening of *w* ≈ 0.1 mm) due to the gauge-length elongation caused by the change on its curvature. Hence, the third and final filter was triggered, and the algorithm sent an alarm about this unexpected event. The sudden event of an unusual peak-to-peak amplitude associated with the loss of linearity could indicate structural damage, such as the rupture of a prestressed tendon caused by, e.g., the passing of an over-weighted truck or corrosion in the tendons. The follow-up mode drift suggests that the gauge-length abruptly changed, which is a good indicator that a crack opening or an unusual relative displacement between the sensor’s anchoring occurred.

**Figure 13:**
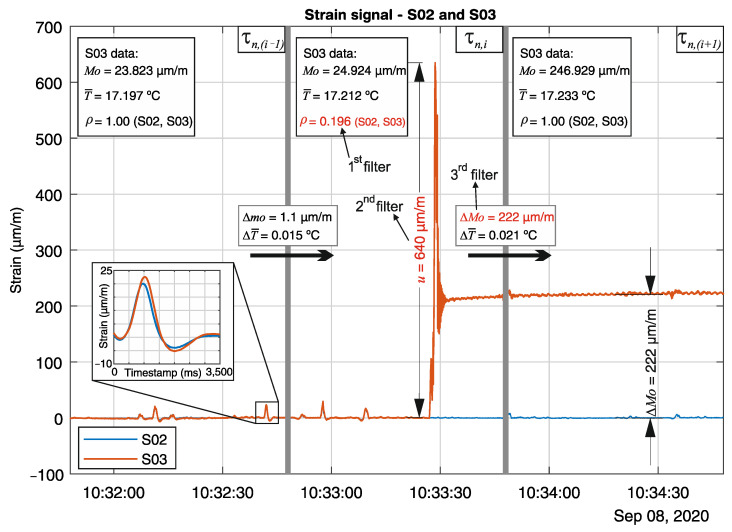
Strain signals for sensors S02 and S03 during an alarm triggering event. The zoom window at about 10:32:40 shows the strains caused due to the regular passing of vehicles.

To better understand the unexpected event depicted in Figure 12e, the strain data from sensors S02 and S03 for the three consecutive one-minute time windows $\tau_{n,\left(i-1\right)}$, $\tau_{n,i}$, and $\tau_{n,\left(i+1\right)}$ (timestamps and duration in Table 2) is shown in Figure 13. For each time window, the statistical strain mode *Mo*, and the mean temperature $\bar{T}$ for sensor S03 is shown, as well as the correlation coefficient *ρ* between sensors S02 and S03 (CF_S02_S03). The statistical parameters are summarized in Table 3. The strain signals are depicted after removing the strain offsets, which is done by subtracting the strain mode from the raw strain signal.

During the time window $\tau_{n,i}$, the unexpected event in the sensor S03 signal begins at timestamp 10:33:27 h. It can be observed that the sensor S03 strain signal displays unusual behavior, which deviates from its neighbor sensor S02. The correlation coefficient identifies the disagreement between sensors S02 and S03, with *ρ* = 0.196 at $\tau_{n,i}$, therefore triggering the first filter (*ρ* < 0.9) of the three-step real-time analysis. Next, the second filter examines the peak-to-peak strain amplitude *u* during $\tau_{n,i}$. A maximum *u* of 640 µm/m is recorded, thus triggering the second filter (*u* > 60 µm/m). Finally, the last filter comes into action, where the sensor S03 strain mode variation Δ*Mo* and mean temperature variation $\Delta \bar{T}$ between the previous and the subsequent time windows are verified. It can be observed that the strain mode and temperature variation between the previous time windows $\tau_{n,\left(i-1\right)}$ and $\tau_{n,i}$ were $\Delta Mo_{i,\left(i-1\right)}$ = 1.1 µm/m and $\Delta {\bar{T}}_{i,\left(i-1\right)}$ = 0.015 K, which is not sufficient to trigger the third filter (Δ*Mo* > 25 µm/m and Δ*Mo* > $15\;µm/m\cdot K\times \Delta \bar{T}=0.225\;µm/m$). However, after the strain perturbation in sensor S03 at $\tau_{n,i}$ takes place, the strain mode variation in the subsequent time window $\tau_{n,\left(i+1\right)}$ detects the new signal offset for sensor S03, caused by the elongation of its gauge-length. The strain mode and temperature variation between the subsequent time windows $\tau_{n,\left(i+1\right)}$ and $\tau_{n,i}$ were $\Delta Mo_{\left(i+1\right),i}$ = 222 µm/m and $\Delta {\bar{T}}_{\left(i+1\right),i}$ = 0.021 °C, finally passing the third and last filter and triggering the alarm call.

**Figure 14:**
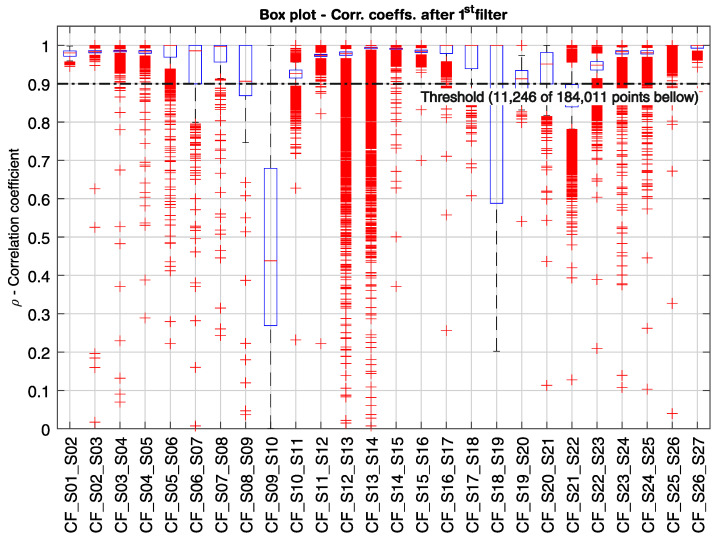
Box plot for the correlation coefficients from sensors S01–S27—1st filter: *ρ* < 0.9.

**Figure 15:**
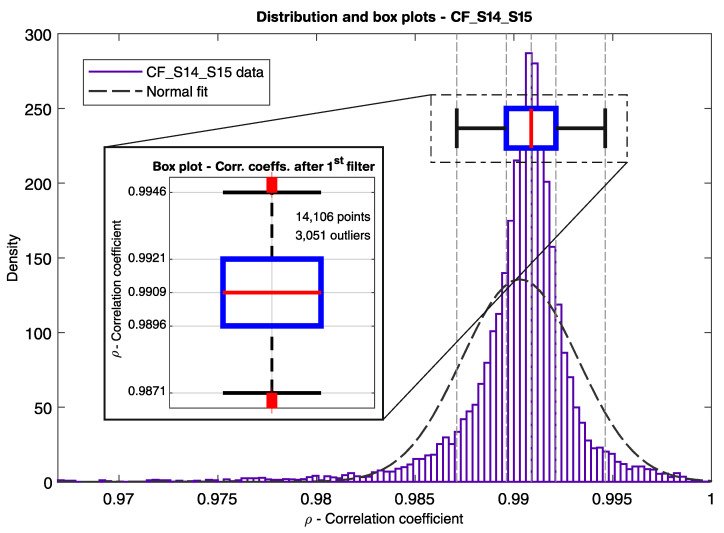
A closer look at the correlation coefficient CF_S14_S15 extracted from Figure 14—density distribution and box plot.

**Figure 16:**
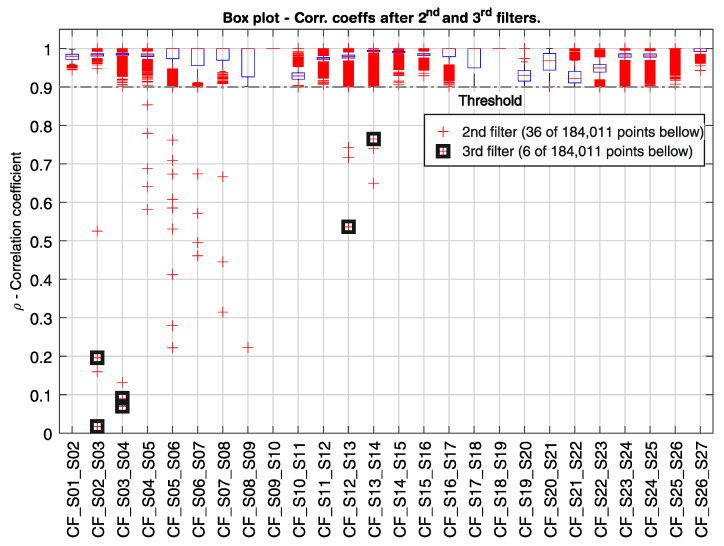
Box plot for the correlation coefficients from sensors S01–S27—2nd filter: *u* > 60 µm/m, and 3rd filter: Δ*Mo* > 25 µm/m and Δ*Mo* > 15 × $\Delta \bar{T}$.

In Figure 14, Figure 15 and Figure 16, the correlation coefficients for every pair of neighboring sensors from S01 to S27 are represented in box plots for the period from 14 July to 6 November 2020 (116 days). In Figure 14, the first filter is displayed as a threshold line at *ρ* = 0.9. From a total of over 184,000 measured time windows $\tau_{n}$, 11,246 points were below the threshold. Although about 94% of the measuring points did not pass the first filter, there would still be many alarms, if only the first filter were used, triggering about 97 alarms per day. Figure 15 shows in detail the correlation coefficient between sensors S14 and S15, where the distribution density, as well as the enlarged box plot, are depicted. It can be noticed that the mean and median values are close to one for well-correlated sensors, and the data dispersion is small. When the second filter is applied (*ρ* < 0.9 and *u* > 60 µm/m), as shown in Figure 16, 36 points remain below the threshold. Nonetheless, there would be an alarm triggered every three days, considering just the first and the second filters. Finally, when the third filter level is engaged, only six points remain Figure 16). Four out of the six remaining points are related to the demonstration described earlier, where the small weight was hanged on the sensor S3’s gauge-length, provoking a perturbation in its strain signal and thus generating two alarms between sensors S02 and S03, and two alarms between sensors S03 and S04. The other two outliers are associated with the correlation coefficients CF_S12_S13 and CF_S13_S14.

From Figure 14, it is seen that the sensors located along the spans—namely sensors S01–S06, S10–S18, and S22–S27—are linearly correlated with their neighbor sensors, having medians and narrow interquartile ranges above 0.9 for the correlation coefficient, and a small number of outliers. The similar behavior can be observed between sensors S07–S09 and S19–S21, located on the massive cross-section at the intermediate supports. However, the first quartile of the correlation coefficients CF_S08_S09 and CF_S19_S20 is smaller than 0.9. Yet, the correlation coefficients for the sensors located at the transition between the section with hollow-cores and the massive cross-sections (Figure 2) have a low correlation level. The coefficient CF_S09_S10, e.g., has a median of 0.44 and dispersed data, which can be seen from the wide interquartile ranging from 0.27 to 0.68. The low correlation level at locations, where the structure’s flexural rigidity changes abruptly are expected on statically indeterminate systems, since the internal loads and deformations distributions are a function of the structural rigidity. 

It can also be observed from Figure 14 that the correlation coefficients CF_S12_S13 and CF_S13_S14 have a large number of outliers when compared with the other sensors located at the spans, with 4137 outliers out of 14,740 points (28%), and 3,590 outliers out of 15,187 points (24%), respectively. Not only is the number of outliers high, but they are in a great quantity smaller than 0.9. While the correlation coefficients for sensors S02–S04, e.g., had only 0.156% of their outliers smaller than 0.9 (Figure 12a), the same rate goes up to 5.163% for the correlation coefficients CF_S12_S13 and CF_S13_S14. Even though the number of outliers with values smaller than 0.9 is high, both correlation coefficients still have a median close to one and narrow interquartile ranges, suggesting a continuous structural dynamic behavior along with sensors S12–S14. Moreover, the low correlated points were always related to the strain signal from sensor S13, either due to a sudden drift in its offset signal or to a low-frequency vibration event after a vehicle’s crossing. This behavior could indicate that the segment covered by the sensor S13 has a higher level of cumulative degradation than its neighbor sensors. Nonetheless, only two points related to sensor S13 triggered the three-step real-time analysis, as shown in Figure 16. 

### 5.2. PCA Post-Processing

**Figure 17:**
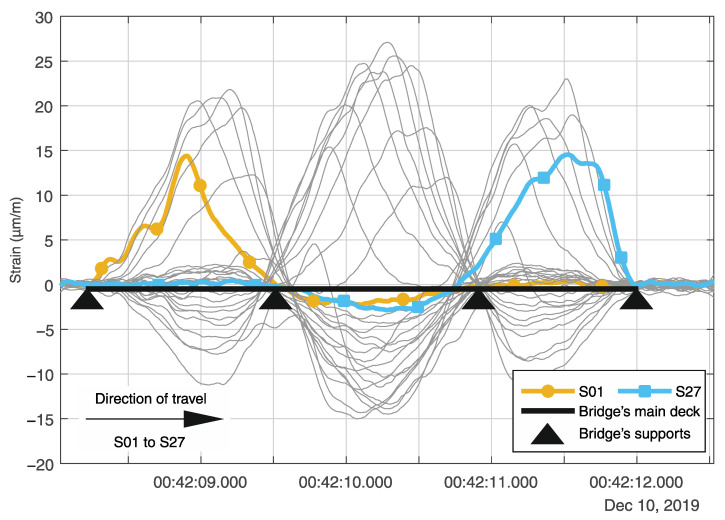
Strain lines for sensors S01 to S27 during the crossing of a heavy vehicle. The data has a length of 1000 observations (5 s). The abutments’ approximated position and the intermediary columns are represented with triangles, and the bridge’s main deck is depicted as a thick horizontal line. The strain lines for sensors S03 and S14 are highlighted. The other strain lines are shown in grey.

The principal components analysis (PCA) is performed to evaluate each sensor’s contribution to the system’s behavior and how they intercorrelate during the crossing of vehicles. A single vehicle’s crossing is analyzed to demonstrate how the PCA works and how the results are interpreted. The example event took place on 10 December 2019 and comprises a time window $\tau_{n}$ with 5 s (1000 samples per sensors) of measurement data during a heavy vehicle crossing in the northern direction. The longitudinal sensors are divided into two groups for analysis according to their driving lanes location. Group A includes sensors S01–S27, and group B contains sensors S28–S54 (Figure 3). The results will be demonstrated only for group A strain lines, as shown in Figure 17.

First, the data set must be organized into a single matrix for the analyzed time window $\tau_{n}$, where each column corresponds to a sensor *S_i_*, and each line to an observation *t_n_*, where *i* is the sensor’s number and *n* the data sample, as follows:

(5) $$S\left(\tau_{1000}\right)={\left(\begin{array}{ccc} S_{1}\left(t_{1}\right) & \cdots & S_{27}\left(t_{1}\right)\\ \vdots & \ddots & \vdots \\ S_{1}\left(t_{1000}\right) & \cdots & S_{27}\left(t_{1000}\right) \end{array}\right)}_{27\;\times \;1000},\;\mathrm{for}\;i\;=\;1\;\mathrm{to}\;27\;\mathrm{and}\;n\;=\;1\;\mathrm{to}\;1000.$$

After normalizing the data matrix, the covariance matrix is constructed, and its eigenvalues and eigenvectors are obtained. There are many eigenvectors as there are variables, and each eigenvector has many elements as the number of variables. Hence, the eigenvectors form a 27 × 27 matrix, given that group A has 27 sensors. 

**Figure 18:**
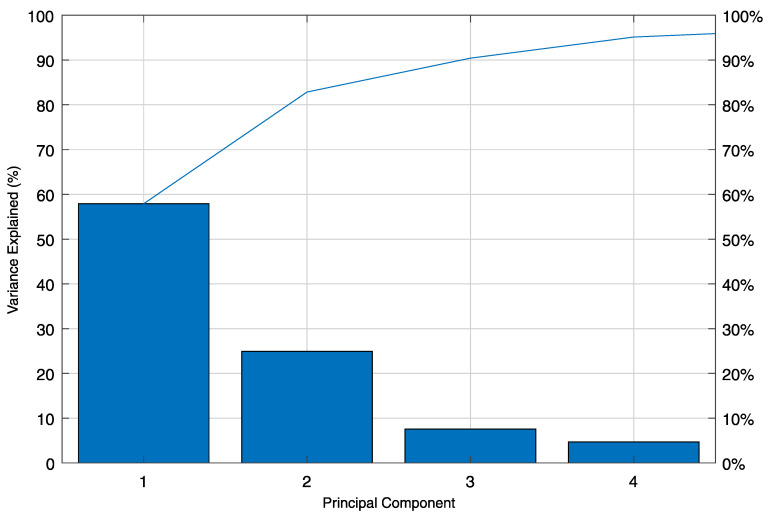
Scree plot of the principal components that explain 95% of the total variance.

The principal components (PC) matrix is obtained by sorting the eigenvectors by their eigenvalues in decreasing order, where the first few principal components explain most of the data variance. Figure 18 shows a scree plot with the decreasing rate at which the PCs explain the variance. The first four PCs explain 95.14% of the total variance, the first and the second being responsible for 82.87%.

**Figure 19:**
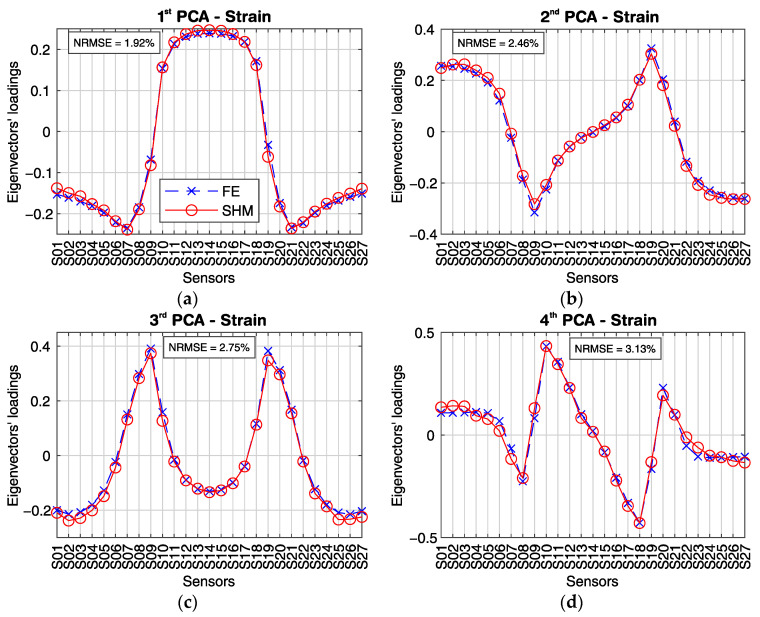
Line-plots of the first four principal components loadings. The coefficients estimated from the SHM measured data are plotted with circle markers and full-line. In contrast, the coefficients calculated from a calibrated FE model are plotted with x-markers and dashed lines. The NRMSE is given for each plot: (**a**) First principal component; (**b**) second principal component; (**c**) third principal component; (**d**) fourth principal component.

Figure 19 shows line plots for the first four PCs’ elements for each sensor in the analyzed group A (S01 to S27). From now on, the elements of a PC will be designated as loading. The calculated loadings from the SHM data are plotted with circle markers and full-line, while the loadings estimated from a calibrated FE model are plotted with x-markers and dashed lines. The normalized root mean square error (NRMSE) between the FE model results and the SHM measured data is 1.92% for the first PC; 2.46% for the second PC, and the second PC; 2.75% for the third PC; and 3.13% for the fourth PC. 

It can be noted from Figure 19a that sensors S10–S18, located in the mid-span, have positive loadings for the first PC, while all sensors located in one of the side spans or in the columns’ region have negative loadings for the first PC. In addition, the line plot for the first PC loadings is symmetric with respect to a vertical axis at sensor S14. Loadings with the same signal mean that their variables are directly correlated, while variables that have loadings with different signals are inversely correlated. Likewise, variables with higher absolute loadings have a more considerable variance than variables with absolute smaller loadings. 

For example, the first PC loadings for sensors S03 and S13 are −0.17 and 0.24, respectively. Since the original data set is composed by the longitudinal strain measurements during a vehicle’s crossing, and we analyze the first PC, it is likely that the strain lines’ behavior from S03 and S13 is inversely correlated. In fact, it can be seen from Figure 17 that the strain lines S03 and S13 always have opposite signals and opposite inclinations. Moreover, the absolute loading value for sensor S13 is larger than for sensor S03, reflecting the more considerable variance of sensor S13.

The second PC (Figure 19b) is most likely related to the shear deformation during the vehicle’s crossing. The second PC’s loadings have absolute maximal values at the intermediary columns (≈0.30) and close to the southern and northern abutment (≈0.25), where the shear force is usually larger. Unlike the first PC, the second PC’s line plot is symmetric about an origin defined by the horizontal zero-line and a vertical line at sensor S14. When the vehicle approaches sensors S08 and S09 at the first column, for example, the shear force reaches its maximal absolute value at that location. In contrast, the shear force at the second column (sensors S19 and S20) is close to zero at the same timestep (since the first column absorbs the shear load as the vehicle is standing on it). Likewise, as the vehicle approaches the sensors S19 and S20 at the second column, the shear force at the second column will reach its maximal value, while the shear force at the first column tends to zero at the same moment. The second PC’s loadings at the two described regions have approximately the same absolute value of 0.30, but with opposite signs. Furthermore, the summation of the second PC’s loadings is zero, just as the summation of the shear energy for a moving load crossing the structure should also be.

Although the third and fourth PCs (Figure 19c,d) explain together only 12.27% of the system’s variance, their loadings are consistently distributed and resemble the bending moment diagram (third PC), and the shear force diagram (fourth PC) for a static uniform distributed loading.

**Figure 20:**
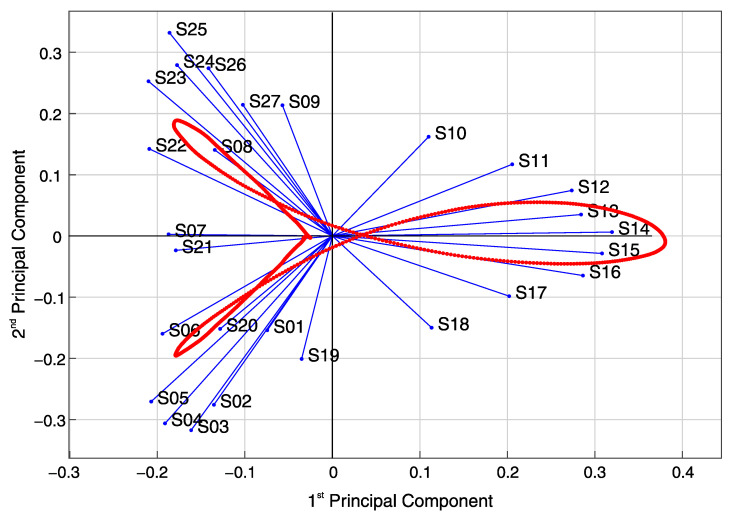
Bi-plot of the first and the second principal components. The vectors represent the contribution of each variable to the PCs, and the red dots are the observations’ scores about the PCs axis.

Figure 20 shows a bi-plot, where the loadings of the 27 sensors for the first two PCs are plotted as vectors in the new coordinate system formed by the first and second PCs axis. The direction and length of the vectors indicate how each variable contributes to the two PCs. The variables’ vectors are systematically distributed in the bi-plot’s quadrants. Sensors S01 to S06 located at the bridge’s first span are in the second quadrant, sensors S22 to S27 located at the third span are in the third quadrant, sensors S10 to S18 located at the mid-span are symmetrically distributed about the first PC axis in the first and fourth quadrants. Sensors S07 to S09 and S19 to S21 located at the massive section at the columns are in the third and second quadrants, respectively.

Figure 20 shows each observation’s scores for the first and second PCs as red dots. The scores are a linear combination of the variables at each observation, weighted by their respective loadings, and then scaled with respect to the maximum vectors’ length. Since there are 1000 observations (1000 samples per sensor in the original dataset), there are 1000 pair of scores with scaled coordinates in the first and second PCs axis. The scores’ path is related to the vehicle’s movement and indicates the direction of most variance after each observation. As it can be seen, the first score is located close to the origin. As the vehicle enters the bridge, the scores’ path enters into the second quadrant, moving in the same direction as the vectors for sensors S01–S06. At some point, the scores’ path changes its direction towards the origin as the vehicle closes to the first column. After the vehicle enters the mid-span, the scores move inside the fourth quadrant, where sensors S10–S13 are located. When the vehicle reaches the bridge’s midpoint at sensor S14, the scores’ path changes once again its direction towards the origin inside the first quadrant, where sensors S15–S18 are. Lastly, the scores’ path moves in the same direction as the vectors for sensors S22–S27 in the third quadrant, and changes its directions towards the origin as the vehicle moves to the end of the bridge.

Finally, the same correlation coefficients calculated by the novel real-time analysis algorithm (as explained and demonstrated in Section 4.1 and Section 5.1) can be extracted from the covariance matrix of the data matrix $S\left(\tau_{1000}\right)$ defined in Equation (5). Thus, the correlation coefficient between two neighbouring sensors is given by the element $cov\left(s_{i},s_{i+1}\right)$ of the covariation matrix $C\left(\tau_{1000}\right)$ of the data matrix $S\left(\tau_{1000}\right)$, with i=1 to N−1, where *N* is the number of sensors (Equation (4)). The correlation coefficient CF_S02_S03 shown in Figure 12a, for example, is equivalent to the covariance element $cov\left(s_{2},s_{3}\right)$. 

**Figure 21:**
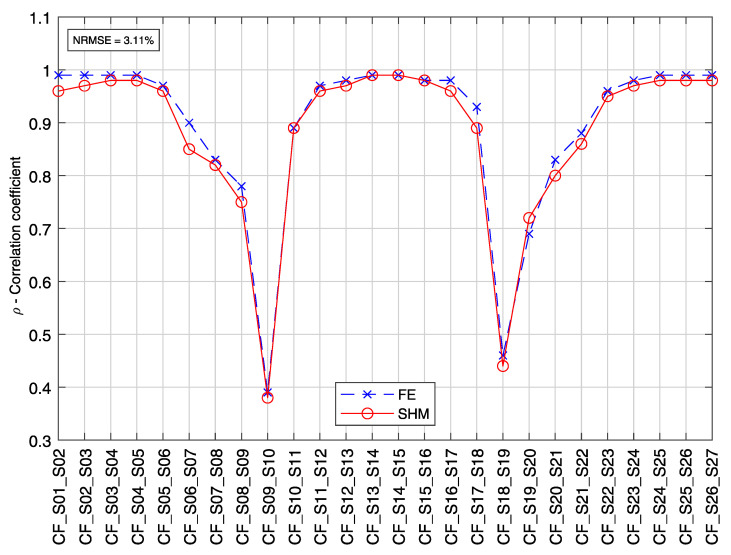
Correlation coefficients from sensors S01–S27 during the crossing of the heavy vehicle defined in Figure 17. The values obtained from the SHM data were calculated by the novel real-time analysis algorithm, while the estimated values from the FE model were extracted from the elements of the covariance matrix $C\left(\tau_{1000}\right)$ of the data matrix $S\left(\tau_{1000}\right)$.

Figure 21 shows the correlation coefficients calculated by the novel real-time analysis algorithm for the heavy vehicle’s crossing, defined in Figure 17. Likewise, the corresponding covariance elements are calculated using the calibrated FE model and plotted for comparison. The calculated correlation coefficients from the SHM measurement data are plotted with circle markers and full-line. In contrast, the estimated values from the calibrated FE model are plotted with x markers and dashed lines. The normalized root mean square error (NRMSE) between the FE model results and the SHM measured data is 1.92%.

## 6. Research Constraints

The deployed monitoring system is a compelling demonstration of a real-case SHM application. The authors sought to address the main weaknesses that restrict the transfer from research to industrial practice. However, some limitations should be noted. The first is the sensors’ placement that prioritizes the longitudinal length coverage, leaving some regions unattended, such as the deck slab’s sides and the columns. The second limitation concerns the absence of a load test to calibrate the system. Although the consistency between the measured data and what the structural theory predicts is undoubted, it is not yet possible to directly relate the measured strains to the crossing vehicle’s actual weight. Moreover, the system operates for approximately one year now, limiting the conclusions about the structure’s behavior to this short period.

## 7. Conclusions

In this work, the authors presented a novel real-time analysis algorithm for detecting unexpected events on a real-sized prestressed concrete bridge, subjected to random-dynamic loads and temperature variation, and a post-processing method based on the principal component analysis (PCA). The methods were demonstrated on a high sensor-count SHM system based on long-gauge FBG sensors installed on a real-sized prestressed bridge in Neckarsulm, Germany. Additionally, the authors describe the data management system developed to enable data condensation and translate the measurement data to reliable and meaningful information.

The proposed real-time analysis’s efficacy was demonstrated by analyzing the history of the three-step validation parameters extracted from the measured data from 14 July to 6 November 2020. First, the three-step validation filtering was described in the example of the data from sensor S03. The steps were:

1. Filtering the correlation coefficients between sensor S03 and its neighbors,
2. filtering relevant peak-to-peak amplitudes, and
3. filtering the strain mode variation,

Leaving at the end one alarm call related to an unexpected event, provoked on sensor S03 during a visit to the bridge. Next, the unexpected event at sensor S03 was shown, where the strain signals from sensors S02 and S03 were plotted for the one-minute time window that trigged the event, explaining in detail the flow of the three-step validation process. Finally, the overall results for all sensors in the quasi-distributed line S01–S27 during the three-step validation process were presented.

Additionally, the strain signals from sensors S01 to S27 were analyzed using the PCA during a heavy vehicle crossing. The first four principal components were used to evaluate the dependency between the strain sensors’ measurements and highlight the correlation coefficient analysis’s reliability to detect behavior changes.

From the results presented in this work, the following conclusions are drawn:

- A robust data management system is essential to handle a high sensor-count monitoring system’s raw data in a real-case SHM application. The data management solution must be able to pre-process the raw data and store it on a reliable database and allow the efficient data selection for post-processing and visualization. Automated scripts should carry out both pre- and post-processing to optimize the speed and the reliability of the data handling. The monitoring system in Neckarsulm generates over 70 thousand measurement points per second, leading to about 600 GB of raw data per month, from which less than 3 GB of meaningful information are permanently stored in the database and are available for post-processing. It would be unbearable to manage such volume of data manually or using traditional spreadsheet software.
- The area coverage and sensor-count are essential aspects to be considered during the development and deployment of a real-size SHM system. A large area of the structure must be measured to correctly depict its behavior and allow the detection of local damages, such as cracks and ruptures in prestressed tendons. The adopted solution with long-gauge FBG sensors offers a comprehensive area-coverage. Local damage can be detected within every two-meter segment along the two quasi-distributed sensor arrays in the longitudinal direction, and within every 1.35-m segment of the five quasi-distributed sensing arrays in the transverse direction. Moreover, the quasi-distributed sensing arrays can be used to analyze the cross-correlation between the sensors to assess how each structure’s segment interacts with one another. This analysis allows the call of unexpected structural behavior and the long-term evaluation of the structural integrity by checking for deviations in the cross-correlation relationships.
- One important contribution of the novel damage detection algorithm is its implementation inside the data acquisition software, enabling the execution of robust analysis during runtime. The data evaluation is performed parallel to the measurements, before data storage and data transfer takes place. Therefore, large amounts of data can be analyzed to detect anomalous behavior as soon as the sensors measure them, without the need and the computational effort to store and transfer thousands of measurement lines for later processing.
- The principal component analysis is a powerful method to reduce large datasets into smaller ones that still hold essential information about the original data. For example, it was possible to reduce a 27 by 1000 matrix of strain measurement points, generated during the crossing of a heavy vehicle, into a 27 × 27 principal components matrix. The first four principal components explain over 95% of the strain data’s variance, and allow an assessment of how each variable contributes to the overall behavior and how they interact with one another. The normalized root-mean-square error (NRMSE) between the PCA of the measured data and the estimated results from the calibrated FE model showed that the monitoring system itself is consistent. The strain measurements are closely and regularly correlated, which endorses the use of correlation coefficients as the critical parameter in the proposed real-time analysis algorithm.

The proposed real-time analysis algorithm is able to address many known limitations of a real-case SHM deployment. First of all, the algorithm runs automatically in real-time during the acquisition software’s runtime without the need for human interference and can detect unexpected changes with low false call rate by the minute. Secondly, the algorithm can tell the unexpected changes’ location with a resolution as small as the sensor’s gauge-lengths in combination with the high sensor-count and quasi-distributed sensing arrays. Moreover, the three-step validation process for alarm triggering is not tied to pre-defined failure modes, absolute limit values, or other known switches. On the contrary, it responds to random and dynamic loads, and it is free from environmental influences, such as temperature variation. 

Another significant point is that the real-time analysis results can be stored and used later for post-processing. The results’ series can then be used to evaluate long-term structural changes. Finally, the proposed algorithm delivers a reliable notification system that allows bridge managers to track unexpected events with valuable information for decision-making.

## Figures and Tables

**Table 1:** Filter’s names and descriptions.

Filter’s Name	Description
first filter	correlation coefficient
second filter	peak-to-peak amplitude
third filter	statistical strain mode variation

**Table 2:** Recorded time windows in Figure 13: Timestamps and duration.

Time Window	Initial Timestamp	Final Timestamp	Duration
$\tau_{n,\left(i-1\right)}$	10:31:48	10:32:48	00:01:00
$\tau_{n,i}$	10:32:48	10:33:48	00:01:00
$\tau_{n,\left(i+1\right)}$	10:33:48	10:34:48	00:01:00

Recorded on 8 September 2020. Time format: hh:mm:ss.

**Table 3:** Statistic parameters for sensors S02 and S03 during the analysed time windows (Table 2).

Sens.	Time Window											
	$\tau_{n,\left(i-1\right)}$				$\tau_{n,i}$				$\tau_{n,\left(i+1\right)}$			
	*u* (µm/m)	*Mo* (µm/m)	$\bar{T}$ (°C)	*ρ*	*u* (µm/m)	*Mo* (µm/m)	$\bar{T}$ (°C)	*ρ*	*u* (µm/m)	*Mo* (µm/m)	$\bar{T}$ (°C)	***ρ***
S02	26.3	5.5	15.2	1.0	27.1	5.6	15.2	0.196	8.1	5.8	15.2	1.0
S03	29.9	23.8	17.2		640.1	24.9	17.2		15.3	246.9	17.2	

## Data Availability

The data presented in this study are available on request from the corresponding author. The data are not publicly available due to ongoing patent registration.
